# Comments on Vimercati et al., 2019, “Asbestos exposure and malignant mesothelioma of the tunica vaginalis testis: a systematic review and the experience of the Apulia (southern Italy) mesothelioma register”

**DOI:** 10.1186/s12940-019-0552-9

**Published:** 2019-12-26

**Authors:** Gabor Mezei, Ellen T. Chang, Fionna S. Mowat, Suresh H. Moolgavkar

**Affiliations:** 1 Exponent, Inc., 149 Commonwealth Drive, Menlo Park, CA 94025 USA; 2 Exponent, Inc., 15735 SE 30th Place, Suite 250, Bellevue, WA USA

**Keywords:** Malignant mesothelioma, Tunica vaginalis testis, Epidemiology, Etiology

We read with interest the review by Vimercati et al. [1] of the scientific literature on malignant mesothelioma of the tunica vaginalis testis (MMTVT). The authors reviewed case series and case reports, and primarily focused their discussion on diagnostic and prognostic characteristics of MMTVT and options for MMTVT treatment. The authors also briefly discussed the potential etiologic role of asbestos exposure in MMTVT development, and declared, “[T]he only causal factor so far ascertained is asbestos exposure, and exposure to different asbestos-containing materials is the only well-documented risk factor, as stated by IARC [International Agency for Research on Cancer].” In this context, the authors referenced our recent review of the epidemiologic literature on mesothelioma of the pericardium and MMTVT [2], stating, “Nevertheless, there are authors [2] who do not agree with the absence, until today, of analytical case-control epidemiologic studies to test this relationship.”

In response to this statement, we note several points. In our systematic literature review and complementary analysis of U.S. registry-based incidence rates of MMTVT [2], we assessed relevant epidemiologic findings regarding the potential etiologic role of asbestos exposure in MMTVT development. We acknowledged the lack of analytical epidemiologic studies, including case-control studies, to test this hypothesis but pointed to several lines of scientific evidence that support our conclusion that the available epidemiologic literature does not support an association, let alone a causal association, between inhaled asbestos exposure and the risk of developing MMTVT. First, in large occupational cohorts with heavy workplace exposures to asbestos, no cases of MMTVT have been reported (e.g., [3]). Second, registry-based incidence rates of MMTVT in the U.S. do not show temporal or geographical trends that would correspond with trends in commercial asbestos use, with due consideration of latency, nor do they reflect incidence rates of pleural malignant mesothelioma, for which asbestos historically played an etiologic role in a substantial fraction of male cases [2, 4]. Third, the incidence of extra-pleural mesothelioma, including MMTVT, in a recent study using the National Mesothelioma Register in Italy does not demonstrate an exposure-response relationship, as MMTVT cases were not reported in some of the highest-risk industries (e.g., asbestos cement, national defense, shipbuilding, and railway industries) [5]. If inhaled asbestos caused MMTVT, the highest risk of MMTVT would be expected in industries with the highest exposure; no such exposure-response relationship is reported (see Mezei et al. [2] for the details of this argument). Interestingly, Vimercati et al. [1] did not reference their Italian colleagues in their article. We also note that case reports and case series are not epidemiologic studies; they cannot establish associations, let alone causal relationships, between exposure and disease. Nevertheless, we found that a substantial proportion of MMTVT cases reported in the published literature did not have documented asbestos exposure [2].

Vimercati et al. [1] also referenced the most recent IARC monograph (2012) on the carcinogenic risks of asbestos [6] in support of their view that asbestos is an established cause of MMTVT. The IARC monograph (2012) does not contain a single mention of MMTVT (or of mesothelioma of the pericardium) either in the main text or in the reference list. Thus, the IARC monograph (2012) does not and cannot conclude that asbestos causes these rare extra-pleural forms of malignant mesothelioma. While an earlier version of the IARC monograph (1987) concerning asbestos mentions MMTVT and pericardial mesothelioma [7], the three references cited in a single sentence are case reports.

In addition, contrary to the opinion expressed by Vimercati et al. [1], there is considerable evidence that in addition to asbestos and some other fibers such as erionite, ionizing radiation increases the risk of malignant mesothelioma (e.g., [8]).

Finally, the current paradigm of carcinogenesis as a process of mutation accumulation implies that all cancers, including malignant mesothelioma can and do occur spontaneously without exposure to any external agents (e.g., [9, 10]).

In summary, we conclude that the available epidemiology provides no evidence that inhaled asbestos exposure is a risk factor for the development of MMTVT. Vimercati et al. [1] provide no evidence to the contrary. We look forward to the results of the forthcoming Italian case-control study mentioned by the authors.

## Data Availability

All references are publicly available.

## Abbreviations

IARC: International Agency for Research on Cancer
MMTVT: Malignant mesothelioma of the tunica vaginalis testis
